# Adjuvant radiotherapy after prostatectomy for prostate cancer in Japan: a multi-institutional survey study of the JROSG

**DOI:** 10.1093/jrr/rrt137

**Published:** 2014-01-01

**Authors:** Manabu Aoki, Takashi Mizowaki, Tetsuo Akimoto, Katsumasa Nakamura, Yasuo Ejima, Keiichi Jingu, Yoshifumi Tamai, Nobuaki Nakajima, Shinya Takemoto, Masaki Kokubo, Hiroyuki Katoh

**Affiliations:** 1 Department of Radiology, The Jikei University School of Medicine, 3-25-8 Nishi-Shimbashi, Minato-Ku, Tokyo 105-8461, Japan; 2 Department of Radiation Oncology and Image-Applied Therapy, Kyoto University Hospital, 54 Shogoin-kawaramachi, Sakyo-ku, Kyoto 606-8507, Japan; 3 Department of Radiation Oncology, National Cancer Center Hospital East, 6-5-1, Kashiwanoha, Kashiwa, Chiba 277-8577, Japan; 4 Department of Clinical Radiology, Graduate School of Medical Sciences, Kyushu University, 3-1-1 Maidashi Higashi-ku, Fukuoka 812-8582, Japan; 5 Department of Radiology, Dokkyo Medical University, 880 Kita-kobayashi, Mibu-cho, Shimotsuga-gun, Tochigi 321-0293, Japan; 6 Department of Radiation Oncology, Tohoku University School of Medicine, 1-1 Seiryo-cho, Aoba-ku, Sendai Miyagi 980-8575, Japan; 7 Department of Radiation Oncology, Tokai University School of Medicine, 143 Shimokasuya, Isehara, Kanagawa 259-1193, Japan; 8 Department of Radiology, Shizuoka General Hospital, 4-27-1 Kita-ando, Aoi-ku Shizuoka 420-8527, Japan; 9 Department of Radiology, Nagoya City University Graduate School of Medical Sciences, 1 Kawasumi, Mizuho-cho, Mizuho-ku Nagoya, Aichi 467-8601, Japan; 10 Division of Radiation Oncology, Institute of Biomedical Research and Innovation Hospital, 2-2 Minatojima Minami-machi, Chuo-ku Kobe, Hyogo 650-0047, Japan; 11 Department of Radiation Oncology, Gunma University Graduate School of Medicine, 3-39-22 Showa-machi, Maebashi, Gunma 371-8511, Japan

**Keywords:** adjuvant radiotherapy, prostatectomy, a multi-institutional survey study (JROSG), SVI invasion, post-operative PSA nadir

## Abstract

In Japan, the use of adjuvant radiotherapy after prostatectomy for prostate cancer has not increased compared with the use of salvage radiotherapy. We retrospectively evaluated the outcome of adjuvant radiotherapy together with prognostic factors of outcome in Japan. Between 2005 and 2007, a total of 87 patients were referred for adjuvant radiotherapy in 23 institutions [median age: 64 years (54–77 years), median initial prostate-specific antigen: 11.0 ng/ml (2.9–284 ng/ml), Gleason score (GS): 6, 7, 8, 9, 10 = 13.8, 35.6, 23.0, 27.6, 0%, respectively]. Rates of positive marginal status, seminal vesicle invasion (SVI) and extra-prostatic extension (EPE) were 74%, 26% and 64%, respectively. Median post-operative PSA nadir: 0.167 ng/ml (0–2.51 ng/ml). Median time from surgery to radiotherapy was 3 months (1–6 months). A total dose of ≥60 Gy and <65 Gy was administered to 69% of patients. The median follow-up time was 62 months. The 3- and 5-year biochemical relapse-free survival (bRFS) rates for all patients were 66.5% and 57.1%, respectively. The GS and marginal status (*P* = 0.019), GS and SVI (*P* = 0.001), marginal status and EPE (*P* = 0.017), type of hormonal therapy and total dose (*P* = 0.026) were significantly related. The 5-year bRFS rate was significantly higher in SVI-negative patients than SVI-positive patients (*P* = 0.001), and significantly higher in patients with post-operative PSA nadir ≤0.2 than in patients with post-operative PSA nadir >0.2 (*P* = 0.02), and tended to be more favorable after radiotherapy ≤3 months from surgery than >3 months from surgery (*P* = 0.069). Multivariate analysis identified SVI and post-operative PSA nadir as independent prognostic factors for bRFS (*P* = 0.001 and 0.018, respectively).

## INTRODUCTION

The primary modality in the treatment of prostate cancer in Japan has long been radical prostatectomy. During the last 10 years, new radical radiotherapy treatment modalities, such as intensity-modulated radiation therapy (IMRT) and brachytherapy, have been introduced and practised generally. Despite the 20% relapse rate for surgical therapy (used in more than half of all cases of prostate cancer), to date most urologists have opted for long-term hormonal treatment. Recognition of the effectiveness of radiotherapy has spread in Japan in recent years, resulting in an increase in salvage radiotherapy. Adjuvant radiotherapy (ADT) following prostatectomy poses advantages for various reasons, but has only been practised in a limited number of hospitals in Japan. This study aggregates the results from post-operative ADT performed at multiple institutions throughout Japan in order to elucidate prognostic factors.

## MATERIALS AND METHODS

**Fig. 1.**
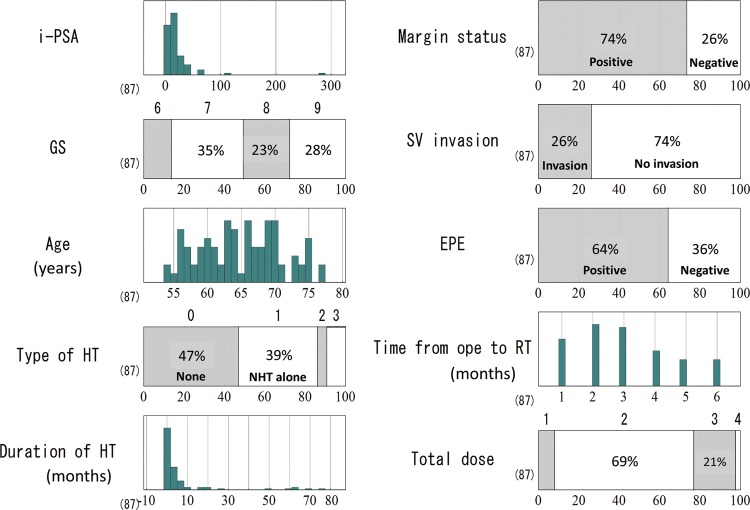
Patient characteristics. Type of HT: 0 (none), 1 (NHT alone), 2 (AHT alone), 3 (NHT + AHT); GS = Gleason score, HT = Hormonal therapy, NHT = Neoadjuvant Hormonal therapy, AHT = Adjuvant Hormonal therapy, SV = Seminal vesicle, EPE = Extra-prostatic extension, ope = operation, RT = Radiation Therapy, TD = Total dose: 1 (TD < 60 Gy), 2 (60 Gy ≤ TD < 65 Gy), 3 (65 Gy ≤ TD < 70 Gy), 4 (70 Gy ≤ TD).

A total of 87 patients, who were given ADT within 6 months following radical prostatectomy in 23 institutions between 2005 and 2007, were enrolled in the JROSG registry office from October 2011 through January 2012. The median patient age was 64 years (range, 54–77), the median preoperative initial prostate-specific antigen (i-PSA) reading was 11.0 ng/ml (range, 2.9–284 ng/ml). The Gleason score (GS) in this study represented the pathologic GS from the surgical specimen, and GS ratios were as follows: 6 (14%), 7 (35%), 8 (23%), 9 (28%) and 10 (0%). The major pathological findings were: positive margin (74%), seminal vesicle invasion (SVI) (26%), and extra-prostatic extension (EPE) (64%). The median post-operative PSA nadir was 0.167 ng/ml (range, 0–2.51 ng/ml), and the median time from prostatectomy to initiation of radiotherapy was 3 months (range, 1–6 months). The most common radiation dose was 60 Gy ≤ Total dose (TD) < 65 Gy (69%), followed by 65 Gy ≤ TD < 70 Gy (21%), < 60 Gy (8%) and 70 Gy ≤ TD (2%). Nearly half of the patients (47%) received no hormonal therapy at all, 39% received neo-adjuvant hormonal therapy (NHT) alone, 5% received adjuvant hormonal therapy (AHT) alone, and 9% received both NHT and AHT. NHT and AHT in this study were administered before and after radiotherapy. The median duration of hormonal therapy was 4 months (range, 0–80 months) (Fig. 1).

Treatment plans for patients in the study were carried out in 6% X-ray simulation (5/87 patients) and 94% computed tomography (CT) simulation (82/87) at 10-MV energy. The irradiated field was the prostate bed alone in 95% of patients (83/87), the small pelvis in 4% (3/87), and the whole pelvis in 1% (1/87). Four-field radiation was used in 35% of patients (31/87), 3D conformal radiation therapy (3D-CRT) in 61% (53/87), and other techniques in 4% (3/87). TD was < 60 Gy in 8% of patients (7/87), 60 Gy ≤ TD < 65 Gy in 69% (60/87), 65 Gy ≤ TD < 70 Gy in 21% (18/87), and 70 Gy ≤ TD in 2% (2/87).

ADT is defined as the administration of radiotherapy to post-prostatectomy patients at high risk of recurrence because of adverse pathologic features prior to evidence of recurrences, regardless of the post-PSA value. The first PSA is generally obtained within 3 months after surgery, and ADT is usually administered within 4–6 months following radical prostatectomy. Disease recurrence after surgery is usually defined as occurring when the detectable PSA level is > 0.2 ng/ml. If patients with a detectable PSA level of > 0.2 ng/ml within 6 months after surgery were given radiotherapy, it was hard to distinguish the divergence between ADT and early salvage radiotherapy. We included these patients in this study.

PSA failure following ADT was defined as PSA ≥ 0.2 ng/ml. If post-operative PSA could not achieve a PSA nadir of <0.2 ng/ml and post-ADT PSA could not achieve a PSA nadir of <0.2 mg/ml once, then the last date of radiotherapy was defined as the date of PSA failure.

Correlations were used to assess the relationships between each prognostic factor. Biochemical relapse-free survivals (bRFSs) were compared for the Kaplan–Meyer method and logrank statistics. Multivariate analysis was performed to analyze the prognostic factors in this study using SPSS^®^ and STATFLEX^®^.

This retrospective study was designed and conducted by the Urologic Oncology Group of the Japanese Radiation Oncology Study Group (JROSG). Also, this study was approved by the IRB of Jikei University and was performed in accordance with the Helsinki Declaration. Informed consent was obtained from all patients prior to radiotherapy.

## RESULTS

**Fig. 2.**
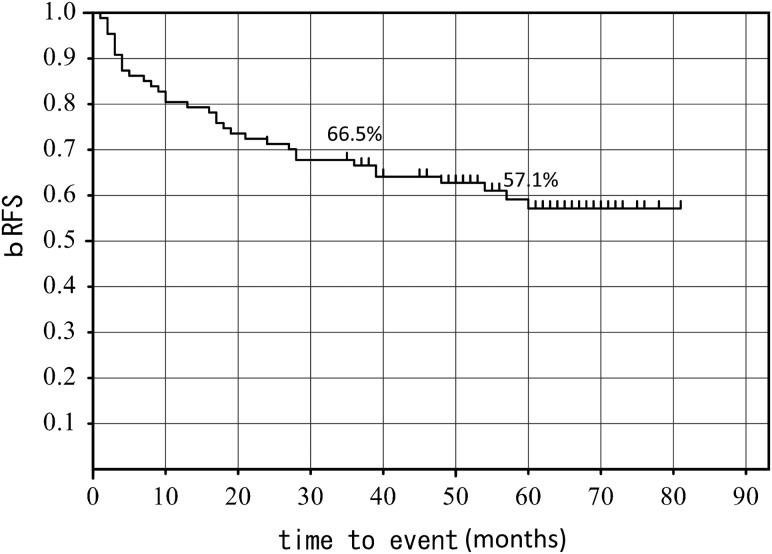
3-year and 5-year bRFS (all cases).

The median follow-up time was 62 months (range, 11–92 months). bRFS rates after 3 and 5 years were 65.4% and 56.5%, respectively (Fig. 2).

### Results of bRFS analysis and correlations for each prognostic factor

There was a significant correlation between GS and margin-positive status (*P* = 0.019) and between GS and SVI (*P* = 0.001), as well as between margin-positive status and EPE (*P* = 0.017). A significant correlation also existed between duration of hormonal treatment and total dose (*P* = 0.026); the total dose was < 60 Gy in significantly more patients receiving long-term treatment with NHT and AHT, while the total dose more frequently administered to more than 60 Gy in patients receiving either NHT or AHT alone (Table 1).

**Table 1.** Correlations for each prognostic factor

		*P* value
Gleason score	Margin positive	0.0198
	Seminal vesicle invasion	0.001
Margin positive	Extra-prostatic extension	0.0174
Type of hormonal therapy	Total dose	0.0261

#### initial-PSA (i-PSA)

The 5-year bRFS rates stratified according to i-PSA scores were as follows: i-PSA < 10, 70.6%; 10 ≤ i-PSA < 20, 50.7%; 20 ≤ i-PSA, 45%. The difference between the 5-year bRFS rate for i-PSA < 10 and for 20 ≤ i-PSA demonstrated a trend towards significance at *P* = 0.083, but there were no significant differences between i-PSA < 10 and 10 ≤ i-PSA < 20 (*P* = 0.14), or between 10 ≤ i-PSA < 20 and 20 ≤ i-PSA (*P* = 0.887) (Table 2).

**Table 2.** 5-year bRFS for each prognostic factor

		*n* (%) total 87	5-year bRFS	*P* value
**i-PSA**	<10	36 (41)	70.6%	
	≥10, <20	29 (34)	50.7%	0.14
	≥20	22 (25)	45%	0.08
**Gleason score**	6	12 (14)	55.6%	0.65
	7	31 (35)	65.3%	
	8	20 (23)	65%	0.69
	9	24 (28)	42.3%	0.07
**EPE**	Positive	56 (64)	58.1%	
	Negative	31 (36)	56.2%	0.95
**Marginal status**	Positive	64 (74)	57.8%	
	Negative	23 (26)	53.2%	0.5
**Seminal Vesicle**	Positive	23 (26)	35.8%	0.01
	Negative	64 (74)	65.1%	
**Total dose**	Low dose	7 (8)	59.7%	
	Intermediate dose	60 (69)	58.3%	0.78
	High dose	20 (23)	42.9%	0.22
**Type of Hormonal therapy**	HT (–)	41 (47)	57.8%	0.35
	NHT	34 (39)	52.7%	0.27
	AHT	4 (5)	50%	0.5
	NHT + AHT	8 (9)	75%	
**PSA nadir**	≤0.2	47 (54)	68.3%	
	>0.2	40 (46)	43.6%	0.02
**Time from surgery to RT**	≤3 months	53 (61)	65%	
	>3 months	34 (39)	43%	0.06

*n* = number of patients, bRFS = biochemical relapse-free survival, i-PSA = initial PSA, EPE = Extra-prostatic extension, HT = Hormonal Therapy, NHT = Neoadjuvant Hormonal therapy, AHT = Adjuvant Hormonal Therapy.

#### Gleason Score (GS)

The 5-year bRFS rates according to GSs were as follows: GS 6, 55.6%; GS 7, 65.3%; GS 8, 65%; and GS 9, 42.3%, showing poor bRFS at GS 6. There was a significant trend between GS 7 and GS 9 (*P* = 0.077), but comparisons between other pairs of GS yielded no significant differences (Table 2).

#### Extra-prostatic extension (EPE)

The 5-year bRFS according to EPE-positive or -negative status showed no significant difference between positive (58.1%) and negative (56.2%) (*P* = 0.954).

#### Marginal status

The 5-year bRFS according to a positive or negative margin showed no significant difference between a positive margin (57.8%) and a negative margin (53.2%) (*P* = 0.58).

#### Seminal vesicle invasion (SVI)

**Fig. 3.**
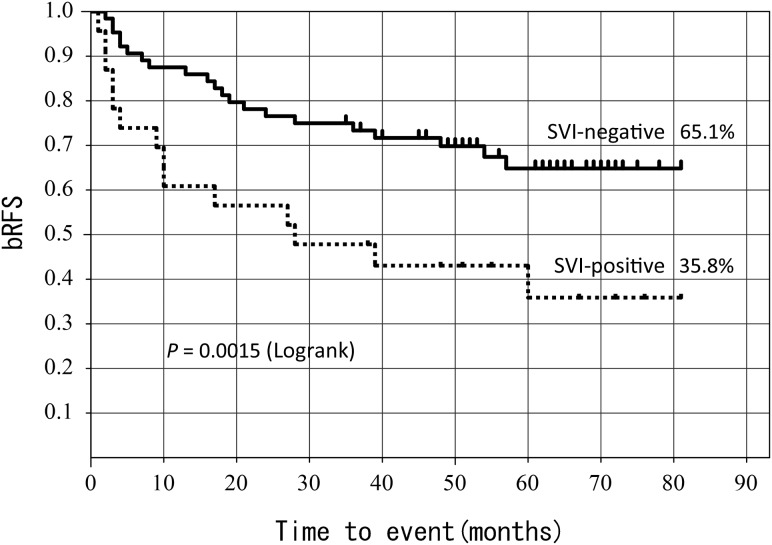
5-year bRFS according to SVI status.

The 5-year bRFS according to SVI showed a significant difference between SVI-positive (65.1%) and SVI-negative (35.8%) (*P* = 0.0015) (Fig. 3).

#### Total dose (TD)

The TD received by patients was stratified into three groups: low dose ( < 60 Gy), intermediate dose (60 Gy ≤ TD < 65 Gy), and high dose (65Gy ≤ TD). The 5-yearbRFS according to TD was 59.7%, 58.3% and 42.9% for low-, intermediate- and high-dose groups, respectively. There was no significant difference either between low dose and intermediate dose (*P* = 0.78), or between low dose and high dose (*P* = 0.22) (Table 2). Concurrent hormonal therapy was more common in the low-dose group, with long-term hormonal therapy (both NHT and AHT) being significantly higher as well at 37.5%.

#### Regimens of hormonal therapy

The 5-year bRFS rate was 75% for long-term recipients of both NHT and AHT, 52.7% for NHT alone, 50% for AHT alone, and 57.8% for patients receiving no hormonal therapy. No significant difference was observed between the groups (Table 2).

#### Post-operative PSA nadir

**Fig. 4.**
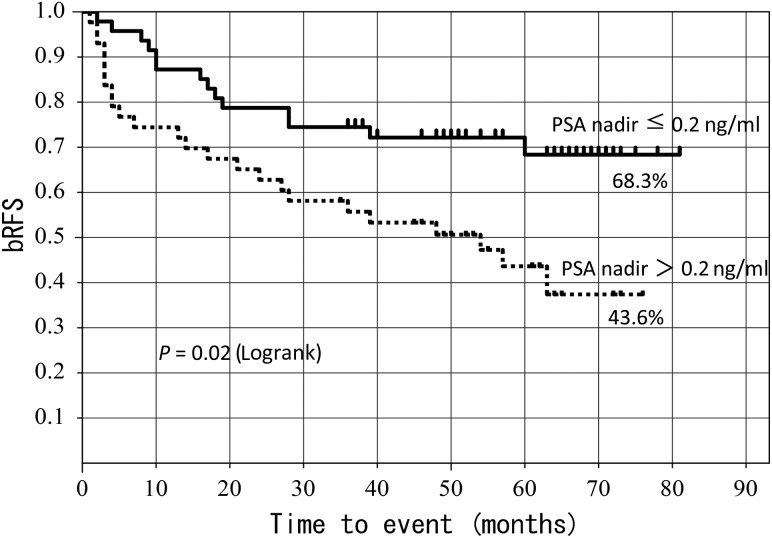
5-year bRFS according to post-operative PSA nadir.

The 5-year bRFS rate was 68.3% for patients with a post-operative PSA nadir of ≤0.2, and 43.6% for those with a post-operative PSA nadir of >0.2, yielding a significant difference (*P* = 0.02) (Fig. 4).

#### Time from surgery to radiotherapy

The 5-year bRFS rate was 65% for patients with three months or less from surgery to radiotherapy, and 43% for patients with more than three months from surgery to radiotherapy.

#### Multivariate analysis

Multivariate analysis of bRFS rates showed that the only significant prognostic factors were SVI (*P* = 0.01, odds ratio 4.781) and post-operative PSA nadir (*P* = 0.0179). There was a trend towards significance in time from surgery to radiotherapy (*P* = 0.0695). Multivariate analysis also identified SVI and post-operative PSA nadir as independent prognostic factors for bRFS (*P* = 0.001 and 0.018, respectively) (Table 3).

**Table 3.** Multivariate analysis of prognostic factors

Prognostic factors	*P* value	Odds ratio	95% CI
i-PSA	0.156		
Gleason score	0.395	1.286	0.72–2.297
Age	0.139		
Hormone therapy (NHT, AHT, NHT + AHT)	0.255	1.70	0.679–4.286
Duration of hormone therapy	0.148		
Marginal status	0.287	0.532	0.167–1.699
Seminal vesicle invasion (SVI)	0.0106	4.781	1.44–15.86
Extra-prostatic extension (EPE)	0.656	1.283	0.427–3.859
Post-operative PSA nadir	0.0179		
Time to EBRT from surgery	0.0695		
Total dose	0.195	1.861	0.727–4.762

i-PSA = initial PSA, EPE = Extra-prostatic extension, HT = Hormonal Therapy, NHT = Neoadjuvant Hormonal therapy, AHT = Adjuvant Hormonal Therapy.

## DISCUSSION

There have been three prospective randomized controlled trials (RCTs) about adjuvant setting for post-prostatectomy compared with surgery only. These three trials each have different patient populations and different primary endpoints. The primary endpoints of SWOG 8794 [1], EORTC 22911 [2] and ARO 96-02 [3] were metastasis-free survival, clinical progression-free survival, biochemical progression-free survival, respectively. The 5- and 10-year bRFS rates were SWOG 8794: 71% and 53%, EORTC 22911: 74% and 60.6%, ARO 96-02: 72% and 61%, respectively. The bRFS rates of these RCTs are better than in our study, in which the 3- and 5-year bRFS rates for all patients were 65.6% and 56.5%, respectively.

We attribute this to differences in patient characteristics. Although percentage of patients with SVI in our study (26.4%) was similar to that of EORTC trial 22911 (25.5%), the percentage of patients with a PSA nadir > 0.2 ng/ml in our data (45.9%) was higher than that of EORTC trial 22911 (28.7%). Similarly, the percentage of patients with GS 8–9 in our data (50.9%) was markedly higher than for those in the ARO96-02 trial (12%) and the SWOG trial (9%). We conclude that patients in our study had higher risk prostate cancer than those in the three RCTs.

### Patient factors

The results of this study concur with previous reports in finding no significant prognostic factors in i-PSA or age. There were also no significant correlations between GS, SVI, EPE, or marginal status. The fact that our study found a significant correlation between GS and SVI (*P* = 0.001), on the other hand the fact that SVI constituted a significant prognostic factor, suggests the importance of GS as well as SVI. Although there is a trend towards significance in the 5-year bRFS between GS 7 and GS 9 (*P* = 0.077), there was no significant difference between the other two groups, and multivariate analysis also failed to demonstrate GS as a significant prognostic factor.

There has been little comparative research on EPE as a prognostic factor for ADT [4]. In this study, the rate of EPE-positive patients was high at 65.6%, and a significant correlation was observed with marginal status (*P* = 0.0174). However, we couldn't find a significant correlation with GS, and there was no significant difference in the 5-year bRFS according to EPE status (*P* = 0.954). In contrast, a significant correlation was observed between marginal status and GS (*P* = 0.0198), although as for EPE there was no significant difference in the 5-year bRFS according to margin status (*P* = 0.508).

In the American College of Radiology's (ACR's) Appropriateness Criteria^®^ [5], the most important prognostic factor stated for biochemical relapse and localized recurrence is marginal positivity, with 40% of margin-positive patients exhibiting elevation of PSA within 5–10 years. ADT also has limited effect on minimal residual tumors, which are not considered poor prognostic factors. Instead, ADT is considered most effective in patients with EPE, SVI, and those with a GS of seven or higher. Some reports have associated elevation of PSA in margin-negative cases with poor prognosis [6].

In this study, the bRFS in SVI-positive patients showed significantly poor prognosis in the past 5 years as compared with that in SVI-negative patients (*P* = 0.01) (Fig. 3). Swanson *et al*. [1] report that radiotherapy significantly improves the 10-year bRFS in patients with SVI compared with observation alone, while Vargas *et al*. [4] also describe a significantly improved 5-year bRFS in SVI-positive patients with ADT. Finally, Taylor *et al*. [7] report results of a multivariate analysis indicating that SVI constitutes the most crucial prognostic factor (*P* = 0.0002), which largely agrees with our own analysis (*P* = 0.01) (Table 3).

### Treatment factors

One intriguing finding of our study is the significant correlation demonstrated between the duration of hormonal treatment and TD. In the low-dose group, hormonal therapy was often performed concurrently in order to compensate for potentially insufficient doses of radiation, and there were significantly more patients receiving long-term treatment with NHT and/or AHT (38%, *P* = 0.026). Although the study yielded no statistically significant differences, our results suggest that one reason for the most favorable 5-year bRFS in the low-dose group was long-term hormonal therapy combined with ADT. A respectable body of research to date has established TD as a significant prognostic factor in curative treatment of prostate cancer, but our study found no such significance. The reason for this may be related to radiotherapy dose; in 2005 the standard TD used for post-operative radiotherapy was 64–66 Gy, while in our study the most common TD was similar at 60 Gy ≤ TD < 65 Gy (69%), followed by 65 Gy ≤ TD < 70 Gy (21%), < 60 Gy (8%) and 70 Gy ≤ TD (2%). Very few patients received doses close to 70 Gy. King *et al*. [8] describe a dose–response curve in salvage radiotherapy that is much less steep than the one described by Hanks *et al*. [9] for curative treatment of prostate cancer. King *et al*. [8] report an expected 3% improvement in bRFS for each additional Gy of irradiation; extrapolating from their dose–response curve, the TD used in our study suggests somewhat lower doses than recent optimal doses.

TDs used in the three randomized controlled trials to date generally utilized relatively low doses in the range of 60–64 Gy, as recommended in the guidelines of the Australian and New Zealand Radiation Oncology Genito-Urinary Group [10]. There are few papers in the literature on dose–response curves in ADT and few non-randomized trials on radiation dose, however Valicenti *et al*. [11] report in their retrospective study of 86 patients that favorable outcomes were obtained at doses of 64.8 Gy and above. Some research [12–14] suggests that ADT may control tumors at lower doses than salvage radiotherapy, but Ost *et al.* [15] describe favorable outcomes with higher doses. They reported that 225 patients with SVI, EPE and positive margins achieved 7-year bRFS rates of 84% and clinical RFS rates of 88% with doses ≥69 Gy, suggesting the effects of increasing doses beyond 68–69 Gy in an adjuvant setting as well. They also describe the role of doses ≥ 70 Gy in IMRT [15]. Harrison *et al*. [16] report that 9-field IMRT allows increasing the dose to up to 72 Gy while maintaining morbidity equivalent to 4-field 3D-CRT of 68.4 Gy.

One other important factor in evaluating radiotherapy is the issue of irradiated field. There is currently no consensus on large-field treatment, including pelvic lymph nodes, even in high-risk patients after radical prostatectomy. There are no randomized trials on radiation fields in adjuvant EBRT or salvage EBRT; the three randomized controlled trials mentioned above all adopted small fields including the prostatic bed, and there are no studies including irradiation of regional lymph nodes. RTOG-0534 included a group with pelvic irradiation, but this group included only patients who received concurrent hormonal therapy.

Spiotto *et al*. [17] studied the results from 114 high-risk (GS ≥ 8, PSA ≥ 20, positive for SVI, ECE or LN) patients (63% receiving whole pelvic radiation and 37% prostatic bed radiation only) out of 160 patients who received either adjuvant EBRT or salvage EBRT. They found that pelvic radiation was effective only for high-risk patients, and there was no significant difference between the two groups of low-risk patients (*P* = 0.9). The 5-year bRFS rate was 21% for patients receiving prostatic bed irradiation only and 47% for those receiving whole-pelvic irradiation. Multivariate analysis showed that whole-pelvic irradiation (*P* = 0.02) and pre-radiotherapy PSA of <0.1 ng/ml (*P* = 0.002) were significant prognostic factors for bRFS.

Wiltshire *et al*. [18] carried out a detailed study of the distribution of surgical clips within the anatomic spatial location of radical prostatectomy and proposed recommendations for radiation fields for post-operative radiotherapy. However, these fields encompass much more target volume than the prostate bed, which has been the primary field used in Japan to date. Thus, irradiated fields used in ADT may be expanded going forward, and IMRT will play a crucial role in ensuring safety at higher dose levels. Ost *et al*. [15] reported on irradiated fields in ADT using IMRT.

**Fig. 5.**
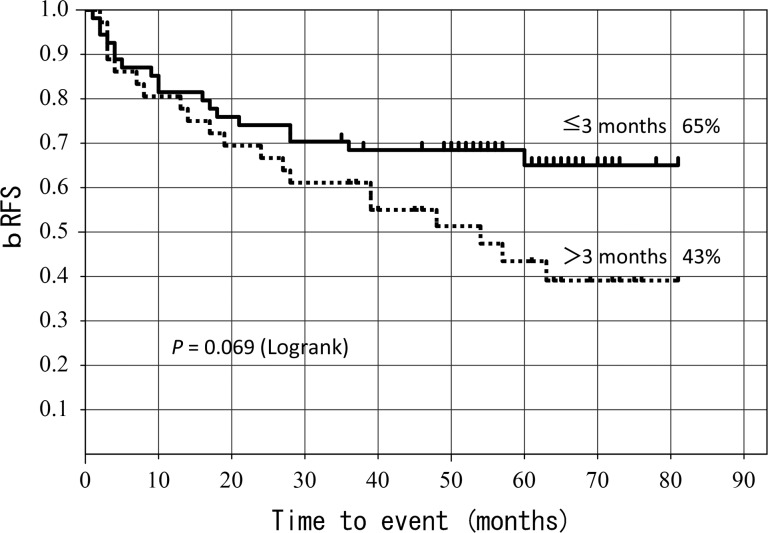
5-year bRFS according to time from surgery to RT.

Our multivariate analysis showed that SVI and post-operative PSA nadir were significant prognostic factors for bRFS. The 5-year bRFS rate was 68.3% for patients with a post-operative PSA nadir of ≤0.2 and 43.6% for those with a post-operative PSA nadir of >0.2, yielding a significant difference (*P* = 0.02) (Fig. 4). A low post-operative PSA nadir is thought to suggest minimal residual tumors, and this assumption is almost in agreement with findings reported by Spiotto *et al*. [17]. The 5-year bRFS rate was 65% for patients with three months or less from surgery to ADT, and 43% for patients with more than three months from surgery to ADT. This is not a clearly significant difference, but it does indicate a trend (*P* = 0.069) (Fig. 5). Both the timing of initiation of radiotherapy and PSA are considered crucial factors [15].

In terms of hormonal therapy combined with ADT, Spiotto *et al*. [17] report that total androgen suppression was effective in conjunction with post-operative radiotherapy only with whole-pelvic irradiation (bRFS: prostate bed only 34.8% vs whole pelvis 52.7%, *P* = 0.0039). Combined hormonal therapy was not recognized as effective in cases of irradiation to the prostatic bed (*P* = 0.45). These interesting results in the adjuvant setting are in agreement with the reports of Roach *et al*. [19] of the efficacy of whole-pelvic irradiation as curative treatment.

Our study found no statistical significance when comparing the arms associated with regimens of hormonal therapy. However, as stated above, the low TD group contained significantly more patients receiving long-term hormonal therapy with both NHT and AHT; this group also demonstrated the most favorable 5-year bRFS, although the difference did not rise to the level of statistical significance. These results suggest that long-term hormonal therapy may compensate for low radiation doses. Corn *et al*. [20] performed a subset analysis of hormonal therapy on 139 high-risk patients enrolled in RTOG 85-01 with EPE and SVI. The 5-year bRFS rate (PSA < 0.5 ng/ml) for patients who underwent adjuvant EBRT (60–65 Gy) was 65% for those receiving combined hormonal therapy versus 42% for those receiving radiation alone. Similarly, King *et al*. [21] showed that concurrent hormonal therapy for patients with GS 8 or higher significantly improved overall survival (*P* = 0.04), with multivariate analysis also supporting concurrent hormonal therapy as being a significant prognostic factor (*P* = 0.0019). Thus, there are great hopes for concurrent and combined hormonal therapy to improve the outcomes of ADT, but we must wait for results from RTOG-0011, RTOG 96-01, and other randomized controlled trials.

## CONCLUSION

We concluded that SVI status and postoperative PSA nadir were significant prognostic factors. Adjuvant EBRT results in relatively favorable outcomes when initiated within three months after surgery in patients with no SVI and a postoperative PSA nadir of 0.2 ng/ml or lower. We support the suggestion that patients with the adverse features listed as follows should be given ADT: (i) multiple positive margins, (ii) EPE, (iii) SVI, or (iv) GS ≥ 8.

However, we still have the following unresolved issues regarding administration of the ADT: (i) optimal radiation fields and techniques, (ii) optimal TD, (iii) NHT vs AHT, and (iv) adverse effects with high dose radiation therapy (≥70 Gy). Regarding these factors we have to await the analysis of the three ongoing RCTs: (i) the GETUG-17 trial (NCT00667069) France, (ii) the TROG RAVES trial (NCT008600652) Australia and New Zealand, and (iii) the MRC-led RADICAL-RT trial (NCT0054107) UK.
